# Supplementary material for: Survival status and its predictors among multi-drug resistance tuberculosis treated patients in Ethiopia: Multicenter observational study
Source: PLoS One. 2020 Nov 9;15(11):e0241684. doi: 10.1371/journal.pone.0241684 (PMC7652297; doi:10.1371/journal.pone.0241684)

Manuscript Click here to access/download;Manuscript;Mortality among MDR-TB patients.docx

Mortality and its predictors among patients receiving Multi-drug resistance tuberculosis treatment in Ethiopia: Multicenter observational study

[1]Asnake Balche Bade, [2]Teshale Ayele Mega

[1]School of Pharmacy, Institute of Health Science, Arbaminch University, Southern Nations, Nationalities and peoples region, Arbaminch, Ethiopia

[2]School of Pharmacy, Faculty of Health Sciences, Jimma University, Oromia regional state, Jimma, Ethiopia

Authors contact address

Teshale Ayele Mega; Email address: tesh.ayu2016@gmail.com

Asnake Balcha Bade; Email address: asnakebalche6@gmail.com

Corresponding author

Teshale Ayele

Email address: tesh.ayu2016@gmail.com

Phone number: +251 91 314 4738

Physical address: Jimma town, Oromia regional state, Jimma, Ethiopia

Abstract

Background: Although substantial progress has been made in combating the crisis of multi-drug resistance tuberculosis (MDR TB), it remained the major global health threat.

Objective: To assess mortality and its predictors among patients receiving multi-drug resistance tuberculosis treatment at selected MDR TB treatment centers of southern and southwestern Ethiopia.

Methods: A retrospective observational study was conducted from April 14 to May 14, 2019, among patients receiving MDR-TB treatment at three MDR-TB treatment centers, Butajira, Arbaminch and Shenengibe Hospitals, located in south and southwestern Ethiopia. A total of 200 records were reviewed using structured questioners. Data was entered into Epi-Data version 4.2.0 for cleaning and exported to SPSS version 21 for analysis. Descriptive analysis was carried out and results were presented by text, tables, and charts. Kaplan-Meier and Cox regression was used to compare baseline survival experience and to determine predictors of treatment outcome respectively. The hazard ratio was used to measure the strength of association and a p-value of <0.05 was considered to declare statistical significance.

Results: Of 200 patients, 54% were males. The mean (± standard deviation) age of the study population was 32.9±9.5years. During follow-up, 22 (11%) deaths were reported making the overall incidence of rate death 11.99, 95% CI [7.89-18.21] per 100,000person-years. The median survival time to death was 375(IQR=249-457) days. Patients with co-morbidity (AHR=23.68, 95% CI [4.85-115.46]), alcohol users (AHR=4.53, 95% CI[1.21-16.97]), and history of poor adherence (AHR=12.27, 95% CI [2.83-53.21]) had a higher risk of death.

Conclusion: In this study, the incidence rate of death was very high. Alcohol use and the presence of co-morbidity were independent predictors of death. Hence alcohol users and patients with comorbidity should be given due attention during therapy.

Key-words: Mortality, MDR-TB, Patients, Ethiopia

Introduction

Multi-drug resistant tuberculosis (MDR-TB) is a major concern at global, regional and country levels. According to the 2019 global TB report, there were 3.4% new cases and 18% previously treated cases of MDR-TB in 2018. In Bangladesh, among reported MDR-TB cases, 1.5% of them were new cases and 4.9% of them were previously treated TB cases. The incident rate of MDR-TB cases in this region was 3.7%. In the Democratic Republic of Congo (DRC), 1.7 % of new cases and 9.5% of previously treated TB cases were reported. The overall incidence of MDR-TB in DRC was found to be 7.2%. Ethiopia ranked 8th among the 30 high MDR-TB burden countries with 2700 MDR-TB cases each year. The estimated prevalence of MDR-TB in the country is 0.71% among newly diagnosed patients and 16% in patients under re-treatment(1).

Moreover, MDR-TB was responsible for a sizeable number of TB-related deaths globally. A study from the United Kingdom reported the death rate of 6.4% (2). According to *Peter et al* MDR-TB was claimed for 3.9% mortality (3). Findings from India and South Africa indicated the mortality rate of 17% (4) and 20 % respectively (5). In Tanzania, 6.5% of mortality was reported among MDR-TB patients (6). Two studies from Ethiopia revealed a mortality rate of 24.4% (16) and 18.3% among MDR-TB patients (7).

The mortality rate due to MDR-TB was amplified by comorbidities(8). The human immune virus (HIV) co-infection was the major risk factor (6,9). In one study, 31.3% of patients have died at 12 months of follow-up. Moderate to severe anemia and being smear positive were significantly associated with death(10). In Ethiopia, the mortality rate of patients was higher in the earlier stages of treatment. Complications, drug-resistance, and smoking had contributed to an increased risk of mortality(11). Though fewer studies had explored the rate of mortality in some parts of Ethiopia, they were single centered and hence, difficult to conclude the incidence

of national mortality. Therefore, this multi-center study was aimed to assess the incidence of mortality and its predictors among patients receiving MDR-TB treatment at selected treatment centers in the south and southwestern regions of the country.

Methods

Study design and setting

A multicenter retrospective observational study was conducted from April 14 to May 14, 2019, among patients receiving MDR-TB treatment at Butajira, Arbaminch and Shenengibe General Hospitals, all located in south and southwestern Ethiopia. They are about 113km, 505km, and 329km respectively away from Addis Ababa, the political center of Ethiopia.

Butajira General Hospital is located at the Gurage zone (southern Ethiopia) and currently serving around 5 million population. It started the MDR-TB treatment service in 2015. Currently, there were 65 MDR-TB patients registered. Of which 49 patients have finished treatment and 16 patients are on treatment. Arbaminch General Hospital is located in the Gamo zone (southern Ethiopia) and currently serving around 6 million population. The Hospital started the MDR-TB treatment service in January 2014. Of 50 patients registered, 45 patients have finished treatment and 5 patients are currently on the treatment. Shenengibe General Hospital is located in the Jimma zone (southwestern Ethiopia) and it is currently serving nearly 5 million people. It started the MDR-TB treatment service by January 2013. Since then, 98 MDR-TB patients were enrolled in the TB treatment programs and those who completed treatment and currently on treatment were 63 and 35 patients respectively.

Study population and patient enrollment

All adult MDR-TB patient charts who fulfilled the eligibility criteria at selected health care facilities were enrolled in the study and there is no special sampling technique employed.

We include all adult patients with the confirmed diagnosis of MDR-TB based on Xpert MTB/RIF® assay (12) and enrolled in the MDR-TB treatment program since January 2013. Charts that reported complete baseline and follow-up data and the intended treatment outcomes were included. We excluded charts of patients with incomplete data and charts of patients transferred to other facilities. Accordingly, 213 charts were assessed for eligibility and 200cahrt were included in the final analysis (Fig. 1)

Data collection procedures and study variables

The data was collected by using a structured checklist prepared from different kinds of literature, WHO guidelines (13,14,15) and national MDR-TB treatment follow up chart. The checklist contains several variables. Patient-related variables include Age, sex, residence, pregnancy, marital status, smoking status, educational level, height, weight, and body mass index (BMI). Disease-related variables such as the category of MDR-TB, drug resistance status, and co-morbidities were also included in the checklist. Furthermore, the checklist also contains drug-related data including type of medication and drug regimen. To capture the impact of time on the treatment outcome, the time at which treatment was initiated and mortality occurred was recorded. All the above data were extracted from patient charts. The data regarding mortality was obtained from the Physicians mortality summary notes as confirmed by the caring physician's name and signature.

Data Quality Assurance

The data collection tool was carefully designed to capture all necessary variables to achieve the study objectives. The charts of each patient were reviewed for inclusion before the data collection. Three clinical pharmacists and three physicians were trained for two days to collect the patient data. The clinical pharmacists collected drug-related information and the patient-related and clinical variables were collected by the physicians. The pharmacists were also

responsible to identify and cross-check adverse drug reactions concerning each anti-TB drug. At each facility, a senior infectious specialist supervised the data collection process. Supervisors followed the data collection process and helped by co-relating diagnostic and laboratory findings with the main outcome. Moreover, a pre-test was conducted on 5% of patients' records to test the effectiveness of the data collection tool and the necessary adjustment was made based on the pre-test findings.

Data processing and analysis

Data were checked for completeness and cleaned using EpiData version 4.2 and exported to STAT-13 for analysis. Categorical variables were summarized by counts, graphs and percentages. The baseline characteristics of the patients were compared using Pearson's chi-square ($\chi 2$). Normally distributed continuous variables were summarized using mean and standard deviation (SD), whereas median and interquartile range (IQR) was utilized to report non-normally distributed continuous variables. The cumulative survival probability of the patients was estimated using the Kaplan–Meier (KM) curve. A bivariate Cox proportional hazard model was first fitted, and variables with p-value <0.25 in the bivariate analysis were further regressed using multivariable Cox proportional hazard model. Crude and adjusted hazard ratios were calculated to estimate the risk of death. On multivariable Cox proportional hazard regression analysis, variables with a p-value of less than 0.05 were considered to declare statistical significance.

Results

Characteristics of the study groups

Of 213 records screened for eligibility, 13 records were excluded and 200 MDR-TB patients' records were included in the analysis (Fig. 1).

Fig 1**:** Sample recruitment chart of patients receiving MDR-TB treatment at selected MDR-TB treatment centers, April 14 to May 14, 2019

Socio-demographic characteristics

The majority, 108(54%) of the patients were males. The mean ± SD age of the study participants was 32.9±9.5. The highest proportions, 78(39%) of participants were Muslims. Most, 111 (55.5%) of them were from rural areas and 99 (49.5%) of the participants were married. The highest proportions have a secondary level of education 74(37%). About 62(31%) of the study participants were merchants. Non-smokers and non-alcoholic comprised 190(95%) and 172(86%) respectively. Baseline smoking status, alcohol consumption and body mass index determined patient status ($p \leq 0.05$) (Table 1).

Table 1: Socio-demographic characteristics of MDR-TB patients in southern and south-west Ethiopia, April 14 to May 14, 2019

Variables	Category	Frequency	Patient status		p-value
			Died (n=22)	Live (n=178)	
Sex	Male<br>Female	108(54%)<br>92(46%)	13(59%)<br>9(41%)	95(53%)<br>83(47%)	0.612
Residence	Urban<br>Rural	89(44.5)<br>111(55.5%)	10(45.5%)<br>12(54.5%)	79(44%)<br>99(56%)	0.924
Smoking status	Yes	10(5%)	4(18%)	6(3.4%)	0.003*
	No	190(95%)	18(82%)	172(96.6%)	
Alcoholic status	Yes<br>No	28(14%)<br>172(86%)	9(41%)<br>13(59%)	19(10.7%)<br>159(89.3%)	p≤0.001*
Marital status	Single<br>Married<br>Divorce<br>Widowed	88(44%)<br>99(49.5%)<br>10(5%)<br>3(1.5%)	9(41%)<br>12(54%)<br>1(2%)<br>1(2%)	79(44%)<br>88(49%)<br>9(5%)<br>2(2%)	0.587
Age	<25<br>26-45<br>>45	50(25%)<br>134(67%)<br>16(8%)	4(18%)<br>15(68%)<br>3(14%)	46(25.5%)<br>124(70%)<br>8(4.5%)	0.179
BMI	<18.5<br>>18.5	55(27.5%)<br>145(72.5%)	10(45.5%)<br>12(54.5%)	45(25%)<br>133(75%)	0.046*

*Statistically significant difference at $p<0.05$

Clinical characteristics and drug-related variables

Pulmonary tuberculosis was the dominant case, i.e. 187 (93.5%) patients. The majority, 156 (78%) of the cases were previously treated/relapse. Thirteen patients (6.5%) had treatment after failure, 22(11%) were new MDR-TB cases and 9 (4.5%) were after loss to follow-up. About 56(26.5%) patients had comorbidity. All patients were tested for HIV infection and 44(22%) were found to be positive for HIV. Diabetes mellitus 9(4.5%) and acute kidney injury 7(3.5%) were among the common co-morbidities diagnosed.

On the drug sensitivity test, samples of 126(63%) patients were resistant to Isoniazid (INH) and 100% of the patients were resistant to rifampicin (RIF). Furthermore, 18(9%) patients were resistant to Ethambutol. Whereas 11(4.5%) and 4(2%) patients were resistant to Streptomycin and Levofloxacin respectively (Table 2).

The median (IQR) hemoglobin and thyroid-stimulating hormone level of the study participants were 14(13-15) g/dl and 5(4-6) μ/ml respectively. Similarly the median (IQR) Serum Creatinine and alanine aminotransferase level were 0.87(0.57-0.98) mg/dl and 34(27-41) IU/L respectively.

According to the baseline clinical and drug-related characteristics described in table 2 below, the presence of comorbidity, experiencing adverse drug reactions, adherence status and resistance to Streptomycin were associated with the patient's status ($p<0.05$).

Table 2: Baseline clinical and drug-related characteristics of MDR-TB patients at selected MDR-TB centers of Ethiopia, April 14 to May 14, 2019

**Variables**	**Category**	**Frequency** (n=200)	**Patient status**		**p-value**
			Died (n=22)	Alive (n=178)	
Site of disease	Pulmonary	187(93.5%)	19(86%)	168(94%)	0.150
	Extra-pulmonary	13(6.5%)	3(14%)	10(6%)	
Treatment group	New	22(11%)	3(14%)	19(10.7%)	
	Previously treated	156(78%)	19(86%)	137(77%)	
	After loss to follow-up	9(4.5%)	0(0%)	9(5%)	0.377
	After treatment failure	13(6.5%)	0(0%)	13(7.3%)	
Comorbidity	Yes	56(28%)	20(90.9%)	36(20%)	p≤0.001*
	No	144(72%)	2(9.1%)	142(80%)	
HIV sero-status	Sero-positive	44(22%)	14(63.6%	30(16.9%)	p≤0.001*
	Sero-negative	156(78%)	8(36.4%)	148(83.1%)	
Diabetes	Yes	9 (4.5%)	4(18.2%)	5(2.8%)	0.001*
	No	191(95.5%)	18(81.8%)	173(97.2%)	
AKI	Yes	7(3.5%)	5(22.7%)	2(1.1%)	p≤0.001*
	No	193(96.5%)	17(77.3%)	176(98.9%)	
Adherence status	Good	173(86.5%)	12(54.5%)	161(90.5%)	
	Fair	20(10%)	6(27%)	14(8%)	p≤0.001*
	Poor	7(3.5%)	4(18.5%)	3(1.5%)	
Taking Vit B6	Yes	196(98%)	20(90.9%)	176(99%)	0.012*
	No	4(2%)	2(9.1%)	2(1%)	
Adverse drug reaction	Yes	57(28.5%)	17(77.3%)	40(22.5%)	p≤0.001*
	No	143(71.5%)	5(22.7%)	138(77.5%)	
Ethambutol	Resistance	18(9%)	4(18.5%)	14(8%)	0.111
	Susceptible	182(91%)	18(81.5%)	164(92%)	
Streptomycin	Resistance	11(5.5%)	4(18.5%)	7(4%)	0.006*
	Susceptible	189(94.5%)	18(81.5%)	171(96%)	
Levofloxacin	Resistance	4(2%)	1(4.5%)	3(2%)	0.366
	Susceptible	196(98%)	21(95.5%)	175(98%)	

AKI: Acute kidney injury, HIV: Human immune virus, *Statistically significant at p-value <0.05

Mortality and predictors

The total analysis time at risk and under observation was 8185days. The overall incidence of death among the population was 11.99, 95% CI [7.89-18.21] per 100,000person years. The first death was recorded 6 months after treatment initiation. The median survival time to death was 375(IQR=249-457) days. There was no statistically significant difference among MDR-TB regimen regarding mortality (Fig. 2)

Fig: 2: Survival estimates of mortality among MDR-TB patients as stratified by initial drug regimen in selected treatment centers of Ethiopia, April 14 to May 14, 2019

A Cox proportional hazard regression analysis was performed to identify predictors of death. Based on this analysis, low hemoglobin level, ADR, comorbidity, smoking, alcoholic status, adherence status and exposure to Vitamin B6 were si [illegible] Log rank p=0.54 [illegible]ted with mortality. On multivariate cox-regression, co-morbidity, alcoholic status, and poor adherence status were independent predictors of death. Consequently, patients who had co-morbidity were 23 times higher hazard of death than patients who had no co-morbidity (AHR=23.68, 95% CI [4.85-115.46]]). Similarly, patients who consume alcohol had 4.5 times higher hazards of death (AHR=4.53, 95% CI [1.21-16.97]. Moreover, poor adherence increased the risk of mortality by more than 12 times (AHR=12.27, 95% CI [2.83-53.21] (Table 3).

Table: 3 Crude and adjusted cox-proportional hazard regression for predictors of death of the cohort, at selected MDR-TB centers of Ethiopia from April 14 to May 14, 2019

Variables		CHR [95%CI]	P-value	AHR [95%CI]	P-value
Smoker	Yes	5.22[1.76-15.53]	0.003	1.26[0.21-7.91]	0.80
	No	1.00		1.00	
Comorbidity	Yes	38.34[8.89-165.27]	p≤0.001	23.68[4.85-115.46]	p≤0.001
	No	1.00		1.00	
Alcoholic users	Yes	5.03[2.15-11.80]	p≤0.001	4.53[1.21-16.97]	0.03
	No	1.00		1.00	

Adherence	Good<br>Fair<br>Poor	1.00<br>6.29[2.34-17.01]<br>14.24[4.51-44.91]	 p≤0.001<br>p≤0.001	1.00<br>1.40[0.40-4.94]<br>12.27[2.83-53.21]	 0.60<br>0.001
Receiving Vitamin B6	Yes<br>No	0.09[0.02-0.41]<br>1.00	0.002	0.40[0.06-2.83]<br>1.00	0.36
ADR	Yes<br>No	9.45[3.48-25.67]<br>1.00	p≤0.001	2.55[0.79-8.20]<br>1.00	0.12
Ethambutol	Resistance<br>Susceptible	2.42[0.82-7.17]<br>1.00	0.11	1.56[0.41-5.88]<br>1.00	0.51
BMI	≤18.5<br>>18.5	2.19[0.95-5.08]<br>1.00	0.07	0.52[0.16-1.63]<br>1.00	0.26
HGB	<12.5gm/dl<br>≥12.5gm/dl	2.23[0.75-6.58]<br>1.00	0.15	1.76[0.39-7.91]<br>1.00	0.46

Good adherence (≥95%), Fair adherence (85-94%), Poor adherence (>85%), HGB: Haemoglobin, ADR: Adverse drug reaction

Discussion

This study summarized the incidence of all-cause mortality and its predictors among MDR-TB patients enrolled in MDR-TB treatment at selected MDR-TB treatment centers of Ethiopia

In this study, the death rate was 22 (11%) making the overall incidence of death 11.99, 95% CI [7.89-18.21] per 100,000person years. The median time to death was 375(IQR=249-457) days. On the multivariate Cox regression presence of co-morbidity, alcohol consumption and poor adherence were independent predictors of death.

In the current study, the overall incidence of mortality was 11.99 per 100,000 person-years. The finding was comparable with a similar study conducted in Lithuania that reported 11 per 100,000 person-per years(16). But lower than the study by Girum et al (17) in which the overall reported incidence of death was 7per 100person-years. The differences might be due to the inclusion of a small number of patients and a shorter follow-up period of the former study. The

reported death rate (11%) in our study was also lower than the other study done in India 17%, South Africa 20%, and eastern Ethiopia 18.3%(11,18,19), but the higher death rate was reported in Peru 5%(8).

In our study, the median time to death was 375(IQR=249-457) days. This was lower than the study from central Ethiopia which reported the median survival time of 480days(11). But much lower median survival time to death was reported by *Kamban et al* (10) in which the median survival time to death was 78(IQR=33.3-154.5) days.

In the current study, patients who had co-morbidity were approximately 24times higher hazard of death (AHR=23.68, 95% CI [4.85-115.46]. As presented earlier in Table 2, HIV/AIDS, diabetes mellitus and acute kidney injury were mostly reported co-morbidities. The presence of such co-morbidities mainly HIV/AIDS (6,9) and diabetes mellitus were strongly related to immune-suppression. Tuberculosis facilitates HIV replication and viral diversification rates through proinflammatory cytokine production. Proinflammatory cytokines increase HIV viral replication and diversity, hence facilitating immune-suppression(20). A study by *Chung-Degado et al* also showed that patients with co-morbidity were 5.4 times higher hazard of death (8). Our finding is also concurrent with the studies conducted in South Africa (5), Tanzania (6), and Ethiopia (7).

The current finding identified alcohol consumption as another predictor of death. Patients who drank alcohol had 4.7 times higher hazards of death (AHR=4.53, 95% CI [1.21-16.97]). Alcohol can increase adverse drug reaction and toxicity to the liver. Moreover, Alcohol consumption detracts general health and may impair immune responses against *M. tuberculosis* (21). Concurrent findings were also generated by *Duraisamy et al,* in which persons who consumed alcohol during treatment had a 4.3 higher hazard of death (AHR=4.3, 95%CI [1.1-17.6] (22). Studies also indicated that an estimated 10% of tuberculosis (TB) deaths are

attributable to problematic alcohol use globally (23). In this study, poor adherence increased the risk of death by almost 12.3 times (AHR=12.27, 95% CI [2.83-53.21]. *Habteyes et.al* reported a similar finding as treatment interruption was significantly associated with unsuccessful treatment outcomes (ARR=1.9; 95% CI [1.4–2.6]) (24).

Our study was not without limitations. Firstly, The retrospective nature of the data source limited us from tracking the major causes of death. Secondly, the method of patients' adherence assessment was also subjective as it is based on patient reports. Third, most of the patients have no data on sputum smear microscopy results. Lastly, but not least, missing patients' income status, wider confidence intervals and inability to screen out the exact causes of death were some of the major hiccups of this study.

Conclusion

In conclusion, this study found a high rate of mortality among patients receiving MDR-TB treatment in the selected settings. Alcohol use, poor adherence, and the presence of co-morbidity were independent predictors of death. This study provided insight into how to provide optimal care of MDR-TB patients with comorbidities, poorly adhered to therapy and habit of alcohol use. However, given all the limitations mentioned above, we urge the readers to interpret the findings of this study cautiously.

Declarations

Ethical approval

The study approved by the Ethical Review Board of Jimma University and given an IRB number of IHRPG1/565/2019. Because of the retrospective anonymous nature of the study, the need for informed consent was waived. Confidentiality was ensured by removing the name and address of the patients from the data.

Consent for publication

Not applicable.

Availability of data and materials

The datasets used and/or analyzed during the current study are available from the corresponding author on reasonable request.

Competing interests

The authors declare that they have no competing interests.

Funding

Not applicable.

Authors' contributions

Authors’ contributions

AB: Conceive the study, collect and analyze the data. **TA**: Conceive the study, analyze the data and draft the manuscript. Both authors have read and approved the manuscript.

Acknowledgments

The authors thank the data collectors and all staff members of the study settings for their valuable contribution. We also would like to thank Jimma University for providing this opportunity to conduct this research.

List of Acronyms and Abbreviations

Cm:	Capreomycin
E:	Ethambutol
Eto:	Ethionamide
MDR-TB:	Multidrug resistance tuberculosis
PAS:	Para-aminosalicylic acid
Pto:	prothionamide
PZA:	pyrazinamide
R:	Rifampicin
RDT:	Rapid diagnostic test
S:	Streptomycin
XDR-TB:	Extensively drug resistance tuberculosis

References

1. WHO. Global tuberculosis report. Geneva: World Health Organization. 2019. *Accessed in April 2019 from* https://www.who.int/tb/publications/global_report/en/
2. Anderson LF, Tamne S, Watson JP, Cohen T, Mitnick C, Brown T, Drobniewski F, Abubakar I. Treatment outcome of multi-drug resistant tuberculosis in the United Kingdom: a retrospective-prospective cohort study from 2004 to 2007. Euro Surveill. 2013;18(40):pii=20601
3. Helbling P, Altpeter E, Egger J, Zellweger J. Treatment outcomes of multidrug-resistant tuberculosis in Switzerland. Eur J Med Sci. 2014;(December):1–7.
4. F. Qazi, U. Khan, S. Khowaja, M. Javaid, A. Ahmed, N. Salahuddin, H. Hussain, M. C. Becerra, J. E. Golub, and AJK. Predictors of delayed culture conversion in patients treated for multidrug-resistant tuberculosis in Pakistan. Union Int Tuberc. 2017; (1):32–8.
5. Marais E, Mlambo CK, Lewis JJ, Rastogi N, Zozio T, Grobusch MP, et al.Treatmentoutcomes of multidrug-resistant tuberculosis patients in Gauteng, SouthAfrica.Infection.2014;42(2):405–13.
6. Mollel EW, Chilongola JO. Predictors for Mortality among Multidrug-Resistant Tuberculosis Patients in Tanzania. J Trop Med. 2017;2017:1–5.
7. Fantaw D, Feyissa M, Hamid S, Shibeshi W. Assessment of the Survival Status and Risk Factors for the Mortality among Multidrug-Resistant Tuberculosis Patients at Adama and Bishoftu General Hospitals, Oromia, Ethiopia : A Retrospective Cohort Study.Adv Pharmacoepidemiol Drug Saf. 2018;7(1):1–5.
8. Chung-Delgado K, Guillen-bravo S, Revilla-Montag A, Bernabe-Ortiz A. Mortality among MDR-TB Cases : Comparison with Drug-Susceptible Tuberculosis and Associated Factors. PLoS-one. 2015; 10(3): 1–10.

9. Padayatchi N. Treatment outcome of drug-resistant Mycobacterium tuberculosis. Int J Tuberc Lung Dis. 18(5)564–570. 2015;14(4):413–9.
10. Berhanu R, Evans D, Rosen S, Sanne I, Long L. High rates of death and loss to follow-up by 12 months of rifampicin-resistant TB treatment in South Africa. PLoS One. 2018;1–13.
11. Molalign S, Wencheko E. Original article Risk factors of mortality in patients with multi-drug resistant TB. Ethiop Heal. 2015;2(1):1–29.
12. Alene K, Viney K, Mcbryde S, Tsegaye A, Clements A. Treatment outcomes in patients with multidrug-resistant tuberculosis in Treatment outcomes in patients with multidrug-resistant tuberculosis in north-west Ethiopia. Trop Dis Med vaccine. 2017;22(3):351-62.
13. Caminero JA. State of the ART Multidrug-resistant tuberculosis : epidemiology, risk factors, and case finding. Int J Tuberc Lung Dis. 2010;14(4):382–90.
14. Caminero JA. Guidelines for Clinical and Operational Management of Drug-Resistant Tuberculosis 2013. 2013. 1-34 p.
15. Putri FA, Burhan E, Nawas A, Soepandi PZ, Sutoyo DK, Agustin H, et al. Body mass index predictive of sputum culture conversion among MDR-TB patients in Indonesia. INT J TUBERC LUNG DIS 18(5)564–570. 2014;18(29):564–70.
16. Williams BG. Tuberculosis country works summary. Indian J Tuberc. 2014;2.
17. Girum T, Tariku Y, Dessu S. Survival Status and Treatment Outcome of Multidrug-Resistant Tuberculosis (MDR-TB) among Patients Treated in Treatment Initiation Centers (TIC) in South Ethiopia : A Retrospective Cohort Study. An MHealth Sci. 2017;331–6.
18. WHO. Global tuberculosis report. Geneva: World Health Organization. 2017 *Accessed in March 2019.*

19. Kalandarova L, Tillashaikhov M, Parpieva N, Saidova S, Gadoev J, Alikhanova N, et al. Treatment outcomes and adverse reactions in patients with multidrug-resistant tuberculosis managed by ambulatory or hospitalized care from 2010–2011 in Tashkent, Uzbekistan. Public Heal Panor. 2016;2(1):21–9.
20. Wang J, Shen H. Review of cigarette smoking and tuberculosis in China : intervention is needed for smoking cessation among tuberculosis patients. BMC Pub Heal. 2009;9 (292):1–9.
21. Myers B, Bouton TC, Ragan EJ, White LF, Mcilleron H, Theron D, et al. Impact of alcohol consumption on tuberculosis treatment outcomes : a prospective longitudinal cohort study protocol. BMC Infect Dis. 2018;1–9.
22. Karthikeyan Duraisamy, Sunilkumar Mrithyunjayan, Smita Ghosh, Sreenivas Achuthan Nair, Shibu Balakrishnan, Jayasankar Subramoniapillai, John E. Oeltmann PK, and Moonan. Alcohol Consumption during Multidrug-resistant Tuberculosis treatment. Ann Am Thorac Soc. 2015;11(5):712–8.
23. Bronwyn M, Tara C, Elizabeth J, Laura F, Helen M, Charles D et.al. Impact of alcohol consumption on tuberculosis treatment outcomes: a prospective longitudinal cohort study protocol. BMC Infect Diseases. 2018; 18:488
24. Habteyes H, Kourosh H, Mohammad A, Mehdi Y, Ephrem T, Zemedu M et.al. intermittent treatment interruption and its effect on multidrug-resistant tuberculosis treatment outcome in Ethiopia. Scientific Reports. 2019; 9:20030

 Click here to access/download;Figure;Fig 1.tif

Fig 1

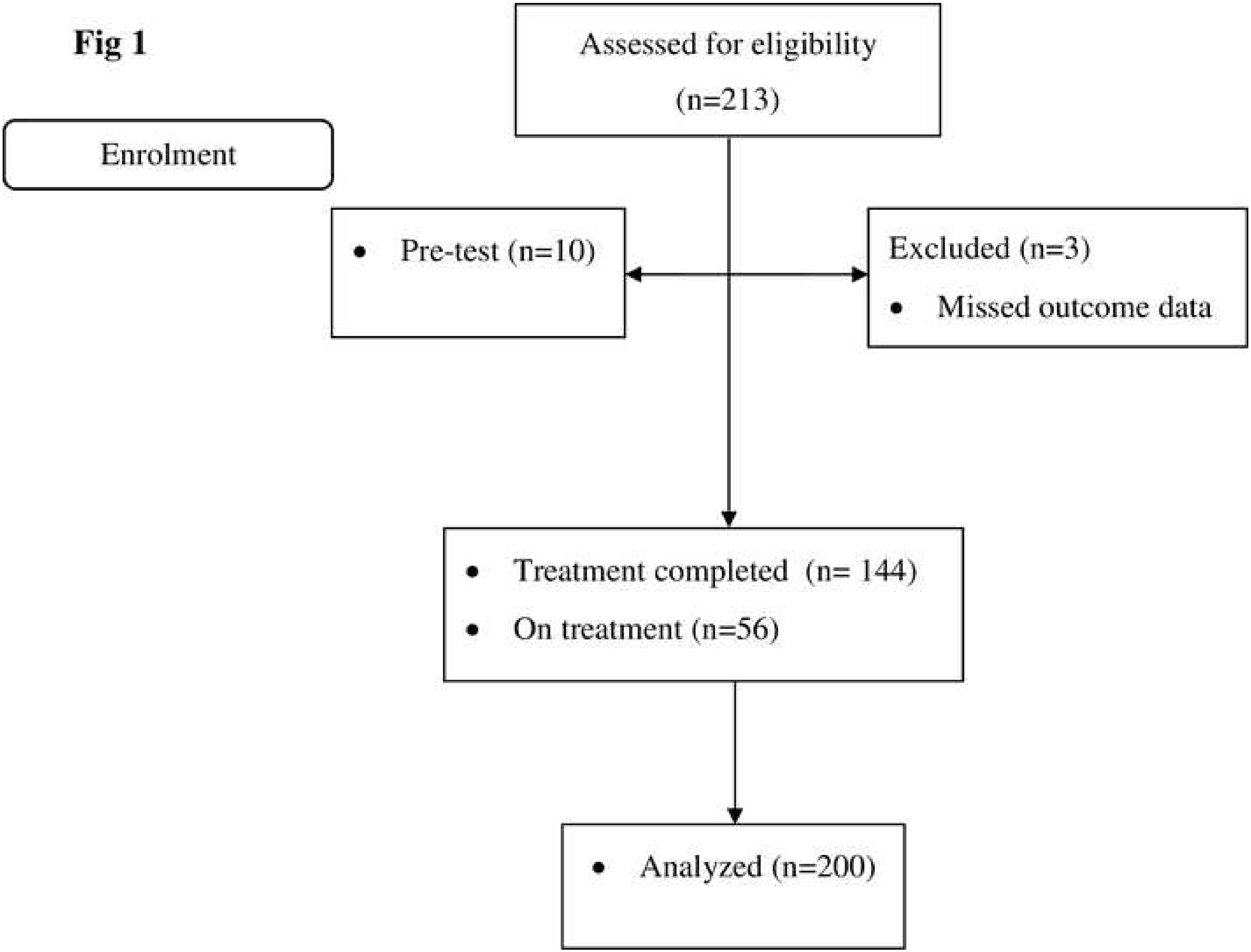

Click here to access/download;Figure;Fig 2.tif

Fig 2

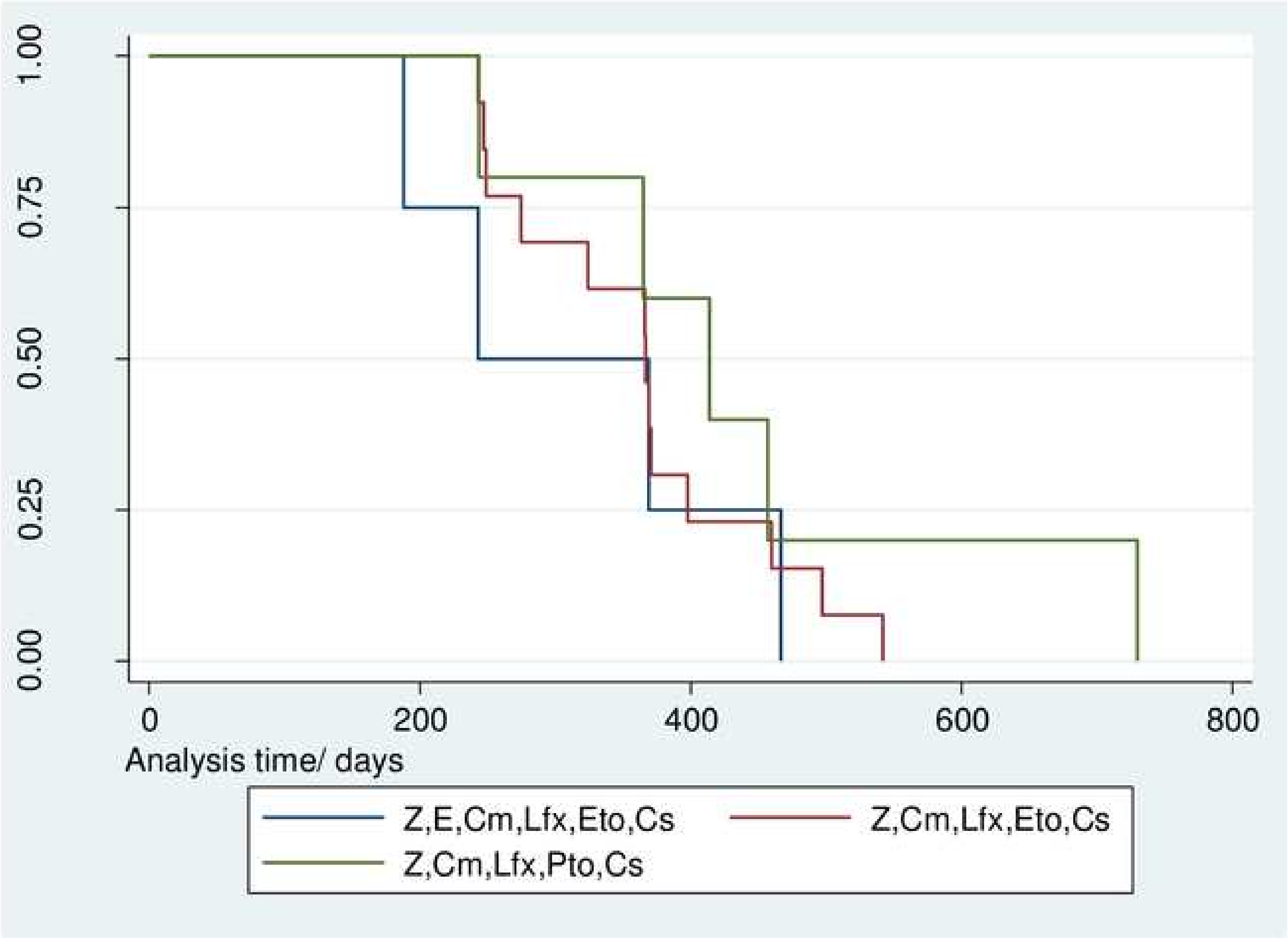

Supplement: Attachment — Submitted filename: 20200614231329.pdf [file pone.0241684.s002.pdf]
